# Supplementary material for: Everlasting impact of initial perturbations on first-passage times of non-Markovian random walks
Source: Nat Commun. 2022 Sep 9;13:5319. doi: 10.1038/s41467-022-32280-6 (PMC9463153; doi:10.1038/s41467-022-32280-6)
Supplement: Supplementary file 1 — Supplementary Information [file 41467_2022_32280_MOESM1_ESM.pdf]

Supplementary Information for: Everlasting impact of initial perturbations on first-passage times of non-Markovian random walks

N. Levernier, T. V. Mendes, O. Bénichou, R. Voituriez, T. Guérin

In this Supplementary information, we provide:

- a characterization of Gaussian processes that have stationary increments at long times (Section A),
- the reason why persistence exponents for such processes cannot be obtained by standard methods (Section B),
- the list of the stochastic processes (and associated simulation algorithms) investigated in this work (Section C),
- a detailed description of the derivation of Eqs. (3), (4), (6) of the main text (Section D), including the description of how θ is numerically obtained (Section D 5).
- a discussion on the restrictions of the theory (Section E),
- a perturbative analysis of our theory around the Markovian Brownian motion ($H \to 1/2$, Section F),
- the analysis of the quenched fBm in the limit of infinite initial temperature (Section G).

A. Characterization of Gaussian processes with stationary increments at long times

We consider a one-dimensional Gaussian random walk $x(t)$ evolving with continuous time $t \geq 0$. Since the process is Gaussian, it is entirely defined by its mean $\langle x \rangle$, which we assume to be constant with time (symmetric walk), and by its covariance $\mathrm{Cov}(x(t), x(t')) = \sigma_0(t,t')$. At long times, we assume that $\sigma_0(t,t')$ admits the scaling behavior:

$$\sigma_0(t,t') \underset{t,t'\to\infty}{\sim} t^{2H}\, G(t/t') \equiv \sigma(t,t'), \tag{S1}$$

where G is a scaling function, H is the Hurst exponent, and the symbol $\sim$ represents the mathematical asymptotic equivalence. The above scaling behavior is standard (see e.g. Ref. [1]). Note also that $\sigma(t,t')$ is the covariance function at long time scales and is defined by Eq. (S1). We will restrict our-selves to $H > 0$ (so that the particle is not trapped at long times at any position of space), and also to $H < 1$ (the long-time process is then non-smooth [1]).

In this work, we study Gaussian stochastic processes that have stationary increments *at long times*, thus displaying *transient* aging. In other words, we assume that the statistics of increments $x(t+\tau) - x(t)$ do not depend on the time elapsed t, if t is large enough. For such processes, one can define a "stationary covariance" σ_s by:

$$\sigma_s(\tau,\tau') = \lim_{t\to\infty} \langle [x(t+\tau) - x(t)][x(t+\tau') - x(t)] \rangle. \tag{S2}$$

For Gaussian processes whose covariance satisfies Eq. (S1), the existence of σ_s requires conditions on the behavior of the scaling function G which we determine now. Using Eq. (S1), we obtain

$$\sigma_s(\tau,\tau') = \lim_{t\to\infty} \left\{ (t+\tau)^{2H} G\left(\frac{t+\tau'}{t+\tau}\right) - t^{2H}\left[G\left(\frac{t}{t+\tau}\right) + G\left(\frac{t}{t+\tau'}\right) - G(1) \right] \right\} \tag{S3}$$

Thus, the quantity σ_s exists when the behavior of $G(u)$ near $u = 1$ is

$$G(u) \underset{u\to 1, u<1}{=} G(1)[1 + H(1-u)] - \kappa_s (1-u)^{2H} + \text{h.o.t}, \tag{S4}$$

which holds for $u < 1$. Here, h.o.t means higher order terms, and κ_s is a positive constant. For $u > 1$ the behavior of G is deduced from the condition that $\sigma(t,t') = \sigma(t',t)$ is symmetric. Note that specifying the linear term (proportional to $1-u$) is not necessary for $H < 1/2$ but it dominates for superdiffusive processes so that it is important in this case. In fact, all correlators we will study in this work satisfy the above condition, irrespectively on the value of H. With the above condition, the stationary covariance reads

$$\sigma_s(\tau,\tau') = \kappa_s \left(|\tau|^{2H} + |\tau'|^{2H} - |\tau'-\tau|^{2H} \right) = \tau^{2H} G_s(\tau/\tau'). \tag{S5}$$

This means that the increments in the future of t have the same covariance function as that of the fractional Brownian motion (fBm), multiplied by a generalized transport coefficient κ_s. Here, G_s is the scaling function associated to the stationary state and is defined by the above equation. The above formula holds also when τ, τ' are negative.

Alternatively, to define the stationary property at long times, we could require that, if we look only at trajectories satisfying the constraint $x(t) = x^*$, and that one looks at the trajectories in the future of this time t, then the statistics of these conditioned trajectories do not depend on t, if t is large enough. In other words, we could define $\sigma_s(\tau, \tau')$ as the limiting value of the covariance of $x(t+\tau)$ and $x(t+\tau')$ *given that* $x(t) = x^*$ *is fixed*, when t is large enough:

$$\sigma_s(\tau,\tau') = \lim_{t\to\infty} \mathrm{Cov}(x(t+\tau), x(t+\tau')|x(t)=x^*) = \lim_{t\to\infty} \left\{ \sigma_0(t+\tau, t+\tau') - \frac{\sigma_0(t+\tau,t)\sigma_0(t,t+\tau')}{\sigma_0(t,t)} \right\}, \tag{S6}$$

where the notation $\mathrm{Cov}(A,B|E)$ represents the covariance of A, B given that the event E is realized, and the second equality is deduced from general formulas on conditional Gaussian processes (see e.g. Ref. [2]). It turns out that the condition for the existence of σ_s defined in (S5) or (S6) is the same and is given by Eq. (S4), and that the two definitions of σ_s are identical. Hence, requiring that the increments of the trajectories are stationary at long time, or imposing a condition on the stationarity of conditional covariances, is actually the same. It is instructive to consider the convergence of the conditional covariance towards the stationary covariance:

$$\mathrm{Cov}(x(t+t_1), x(t+t_2)|x(t)=0) - \sigma_s(t_1,t_2) \underset{t\to\infty}{\sim} -\frac{\kappa_s^2 (t_1 t_2)^{2H}}{t^{2H} G(1)}. \tag{S7}$$

This convergence is therefore algebraic, which was expected due to the absence of characteristic relaxation time.

In summary, in this Section we have characterized the Gaussian processes that have long time stationary increments: these are the Gaussian processes that display a scaling function G satisfying the condition (S4).

B. The reason why standard methods to calculate persistence exponents fail when applied to Gaussian processes with stationary increments at long times

For one-dimensional Gaussian stochastic processes, a standard method to calculate the persistence exponent θ (defined so that $S(t) \propto 1/t^\theta$ at long times) consists in applying the Lamperti transform to obtain a stationary Gaussian process, and then to apply the independent interval approximation (IIA). This method is not exact but gives in general very good estimates of persistence exponents [3]. The Lamperti transform consists in defining logarithmic times $T \equiv \ln t$ and rescaling $x(t)$ by its root mean square to obtain a stationary process, i.e. defining the stochastic process $X(T) \equiv x(t)/[G(1)t^{2H}]^{1/2}$. The covariance function of the transformed process $X(T)$ (still Gaussian) reads

$$\langle X(T_1)X(T_2)\rangle = \frac{t_1^{2H} G(t_1/t_2)}{G(1) t_1^H t_2^H} = e^{-H(T_2-T_1)} \frac{G(e^{-(T_2-T_1)})}{G(1)} \equiv a(|T_2-T_1|). \tag{S8}$$

This correlation function depends only on the difference $T_2 - T_1$, meaning that the process $X(T)$ is stationary. For small positive T, with the hypothesis (S4), this correlation function behaves as

$$a(T) \underset{T\to 0}{\simeq} (1 - HT + ...)[G(1)(1+HT) - \kappa_s T^{2H} + ...]/G(1) = 1 - [\kappa_s/G(1)]T^{2H} + \text{h.o.t.} \tag{S9}$$

For $0 < H < 1$, the above expansion means that $X(T)$ is a *non-smooth* Gaussian process, because smooth Gaussian processes admit a correlation function behaving as $a(T) = a(0) - bT^2 + ...$ for $T \to 0$. Now, the IIA method consists in evaluating the statistics of the time between two successive zeros of a smooth stationary process, but by construction for a non-smooth process this very concept of interval between two zeros is ill-defined. Hence by construction the IIA method can be applied only to smooth Gaussian processes. Therefore it cannot be applied to evaluate persistence exponents in our problems, where the dynamics displays stationary increments at long times. This has been noted in Ref. [3] for the study of persistence in fluctuating interfaces, but what we have shown here is that this restriction is more general and associated to the fact that the process has stationary increments at long times.

C. List of stochastic processes investigated in this work

1. "Quenched" fBm (type I process)

We describe here the stochastic processes for which we will study persistence exponents. We will consider a process which we call "quenched" fBm, for which the correlator at long times is

$$\sigma(t,t') = \sigma_{\rm I}(t,t') \equiv \frac{(1-T)(t+t')^{2H} + T[t^{2H}+t'^{2H}] - |t-t'|^{2H}}{[(1-T)2^{2H}+2T]}. \tag{S10}$$

The function G associated to this correlator satisfies the condition (S4), meaning that it corresponds to a process $x(t)$ that becomes with stationary increments at long times. Depending on H, this correlator corresponds to various physical models (which we used to simulate $x(t)$ for various values of H). For example, in $d=1$, $x(t)$ can be seen as the local height of a one dimensional interface $x(t)=h(s=0,t)$ which obeys the dynamics

$$\partial_t h = -(-\partial_s^2)^{1/(2-4H)} h(s,t) + \xi(s,t), \qquad \langle \xi(s,t)\xi(s',t')\rangle = 2T_1\delta(s-s')\delta(t-t'), \tag{S11}$$

where T_1 is the temperature during the dynamics. Here, we will focus on the cases $H=1/4$ (Edwards-Wilkinson dynamics) and $H=3/8$ (Mullins-Herring dynamics). The correlator (S10) is obtained when $x(t)=h(s=0,t)$ is calculated with an initial condition corresponding to a stationary state of Eq. (S11) with initial temperature $T_1=T$, while the dynamics at $t>0$ takes place at temperature $T_1=1$. Let us recall here how (S10) is obtained from the stochastic equation (S11). Let us denote $z=1/(1-2H)$ and focus on the cases $z=2$ and $z=4$. First we take the spatial Fourier transform of Eq. (S11), with $\hat{h}(q,t)=\int_{-\infty}^{\infty} ds\; h(s,t)e^{-iqs}$. The obtained equation is

$$\partial_t \hat{h}(q,t) = -|q|^z \hat{h}(q,t) + \hat{\xi}(q,t), \qquad \langle \hat{\xi}(q,t)\hat{\xi}(q',t')\rangle = 4\pi T_1(t)\delta(q+q')\delta(t-t'), \tag{S12}$$

where we have considered a time dependent temperature $T_1(t)$. The solution is obviously

$$\hat{h}(q,t) = \hat{h}(q,0)e^{-|q|^z t} + \int_0^t dt' \hat{\xi}(q,t')e^{-|q|^z(t-t')}. \tag{S13}$$

First, we determine the stationary state at a constant temperature $T_1(t)=T$: at long times, we have

$$\langle \hat{h}(q)\hat{h}(q')\rangle_{\text{stat},T} = \lim_{t\to\infty}\int_0^t dt' \int_0^t dt'' \langle \hat{\xi}(q,t')\hat{\xi}(q',t'')\rangle e^{-|q|^z[2t-t'-t'']} = 2\pi T\frac{\delta(q+q')}{|q|^z}. \tag{S14}$$

Next, let us define $x(t)=h(0,t)-h(0,0)$ the stochastic process of interest. For any $t,\tau\geq 0$, its covariance reads:

$$\langle x(t+\tau)x(t)\rangle = \frac{1}{4\pi^2}\int_{-\infty}^{\infty} dq \int_{-\infty}^{\infty} dq' \langle [\hat{h}(q,t+\tau)-\hat{h}(q,0)][\hat{h}(q',t)-\hat{h}(q,0)]\rangle \tag{S15}$$

$$= \frac{1}{4\pi^2}\int_{-\infty}^{\infty} dq \int_{-\infty}^{\infty} dq' \Bigg\{ \langle \hat{h}(q,0)\hat{h}(q',0)\rangle (e^{-|q|^z(t+\tau)}-1)(e^{-|q'|^z t}-1) + \int_0^{t+\tau} dt' \int_0^t dt'' \langle \hat{\xi}(q,t')\hat{\xi}(q',t'')\rangle e^{-|q|^z(2t+\tau-2t')} \Bigg\}, \tag{S16}$$

where we have used (S13). We calculate this quantity by assuming that the initial state is the equilibrium state at temperature T described by equation (S14), and that at $t>0$ the temperature is $T_1=1$. We obtain

$$\langle x(t+\tau)x(t)\rangle = \int_{-\infty}^{\infty}\frac{dq}{2\pi}\Bigg\{ \frac{T}{|q|^z}(e^{-|q|^z(t+\tau)}-1)(e^{-|q|^z t}-1) + 2\int_0^t dt' e^{-|q|^z(2t+\tau-2t')}\Bigg\}. \tag{S17}$$

Performing the integrals leads to $\langle x(t)x(t+\tau)\rangle = \sigma_{\rm I}(t,t')$ given by Eq. (S10), up to a multiplicative factor which does not modify the persistence exponents.

The fact that Eq. (S10) is obtained after a sharp change of temperature in a model of collective dynamics justifies the name "quenched fBm". Interestingly, the correlator (S10) with $T=0$ and $H=1/4$ is also that of a tagged particle in single file diffusion in crowded narrow channels, for which the position of all other particles is initially fixed (which corresponds to the zero temperature state in this problem) [4]. Next, if one replaces $h(s,t)$ by $\mathbf{r}(s,t)$ in Eq. (S11), one

obtains the equation for the dynamics of the positions of monomers $\mathbf{r}(s,t)$ at time t and position s along the chain, for the Rouse chain ($H = 1/4$, a bead-spring chain) or the semi-flexible chain ($H = 3/8$). Other values of H can be obtained for the dynamics of the position $\mathbf{r}_i(t)$ of monomers in hyperbranched (fractal) macromolecules

$$\partial_t \mathbf{r}_i = -\sum_j A_{ij}\mathbf{r}_j(t) + \mathbf{f}(s,t), \qquad \langle f_{i,\alpha}(s,t) f_{j,\beta}(s',t')\rangle = 2T_1\delta_{ij}\delta(t-t')\delta_{\alpha,\beta}, \tag{S18}$$

where α, β are spatial coordinates (x, y, z in $d = 3$) and A_{ij} is the dynamical matrix, with $A_{ij} = -1$ if beads i, j are connected and zero in the contrary, and A_{ii} is the number of connected neighbors around bead i.

Simulations.- In practice, we have simulated processes $x(t)$ (or $\mathbf{r}(t)$ in d dimensions) by integrating numerically a spatially discretized version of Eq. (S11) (for $H = 1/4$ and $H = 3/8$) or Eq. (S18), for which we took the connectivity matrix of a Vicsek fractal of functionality $f = 4$ [5], with $H = \ln(3)/\{2\ln[3(1+f)]\} \simeq 0.203$. Initially the polymer chain (or interface) was prepared at temperature T with a reactive monomer at distance x_0 from the target and we recorded the time to the crossing of $x = 0$ (in $d = 1$), or the time to reach a sphere of radius a in d dimensions. To simulate the dynamic of VFs, we have decomposed the dynamics on eigenmodes

$$\mathbf{r}_n = \sum_i b_i \mathbf{a}_i \ ; \ \partial_t \mathbf{a}_i = -\lambda_i \mathbf{a}_i + \mathbf{f}_i(t), \langle f_i(t) f_j(t')\rangle = 2T_1\delta(t-t')\delta_{ij} \tag{S19}$$

where $\mathbf{r}_n$ is the reactive monomer, λ_i are the eigenvalues of A and b_i the projection of the vector $(0, ..., 0, 1, 0, ...0)$ on the eigenspace associated to λ_i. We used the methods of Ref [6] to identify iteratively the set of (λ_i, b_i). We simulated (S19), which is much more efficient than using (S18) since the number of distinct eigenvalues for fractals is much smaller than the number of beads. In all simulations, we measured θ by plotting the survival probability in log-log scale, including the largest times allowed by the number of accumulated stochastic trajectories. Note that, in particular for low values of θ, the regime of low values of $S(t)$ could not be reached so that the measurement of θ is not very accurate. We checked that the measured values do not depend on microscopic parameters (initial distance to target, target size, time steps, number of monomers...).

2. *fBm constrained on its past (type II process)*

We consider here the statistics of trajectories of a fractional Brownian motion conditioned on its past value. This process is obtained by considering a standard fractional Brownian motion, and asking for the statistics of $x(t)$ for $t > 0$, given that the past trajectory $x(\tau)$ is known exactly for all past times $\tau < 0$. To understand how this process is built, let us consider the case that the process is conditioned on n past times $\tau_i < 0$ (instead of all past times). In this case, general formulas on Gaussian processes (see e.g. chapter 3 in Ref [2]) show that the statistics of the conditioned process can be obtained from the knowledge of the covariance matrix $S_{ij} = \sigma_s(\tau_i, \tau_j)$ (for the unconditioned process, at the times τ_i at which one imposes the conditions):

$$\mathbb{E}(x(t)|[x(\tau_1), ...x(\tau_n)]) = \sum_{i,j=1}^{n} \sigma_s(t, \tau_i)\ (S^{-1})_{ij}\ x(\tau_j), \tag{S20}$$

$$\mathrm{Cov}(x(t), x(t')|[x(\tau_1), ...x(\tau_n)]) = \sigma_s(t, t') - \sum_{i,j=1}^{n} \sigma_s(t, \tau_i)\ (S^{-1})_{ij}\ \sigma_s(t', \tau_j). \tag{S21}$$

The conditional means and covariances can thus be found at the cost of solving a linear equation to find the inverse matrix S^{-1}. In the continuous limit $n \to \infty$, the problem of finding S^{-1} is replaced by an integral equation which was solved by Yaglom (for $H < 1/2$) [7] and Gripenberg and Norros (for $H > 1/2$) [8] (see also [9, 10]), with the result:

$$\mathbb{E}(x(t)|[x(\tau), \tau < 0]) = \begin{cases} \frac{\cos(\pi H)}{\pi} \int_0^\infty d\tau \left(\frac{t}{\tau}\right)^{H+1/2} \frac{x(-\tau)}{t+\tau} & (H < 1/2), \\ x(0) + \frac{\cos \pi H}{\pi} \int dx(-\tau) \int_0^{t/\tau} dv \frac{v^{H-1/2}}{v+1} & (H > 1/2). \end{cases} \tag{S22}$$

Note that in the second line the integration runs over stochastic trajectories, see Ref. [8]. It is clear from Eqs. (S20), (S21) that the formula for the conditional covariance is the same as that for the conditional mean (up to an additive term σ_s) if we replace the past trajectory $x(\tau_i)$ by $\sigma_s(t', \tau_i)$, so that

$$\sigma(t, t') = \mathrm{Cov}(x(t), x(t')|[x(\tau), \tau < 0]) = \sigma_s(\tau, \tau') - \frac{\cos(\pi H)}{\pi} \times \begin{cases} \int_0^\infty d\tau \left(\frac{t}{\tau}\right)^{H+1/2} \frac{\sigma_s(t', -\tau)}{t+\tau} & (H < \frac{1}{2}) \\ \int_0^\infty d\tau \int_0^{t/\tau} dv \frac{v^{H-1/2}}{v+1} \frac{d}{d\tau}\sigma_s(t', -\tau) & (H > \frac{1}{2}) \end{cases} \tag{S23}$$

Note that, in the case $H > 1/2$ in Eq. (S22) we have considered that the integration over the trajectory can be replaced by a sum over $x(t_{i+1}) - x(t_i)$ in discrete time, applying the same linear operator on σ_s leads to the derivative of σ_s with respect to τ in the above equation. Replacing σ_s by its value (S5), we obtain the following formula for the covariance of a fBm conditioned on its past:

$$\sigma(t,t') = \sigma_{\rm II}(t,t') \equiv \kappa_s \left\{ -|t-t'| + t^{2H} + t'^{2H} + \int_0^\infty dx \frac{\cos(\pi H)\ [(t+xt')^{2H} - t^{2H} - (t'x)^{2H}]}{\pi\ x^{H+1/2}(1+x)} \right\}, \tag{S24}$$

which is valid both for $H > 1/2$ and $H < 1/2$. Note that the normalization $\sigma(t,t) = t^{2H}$ is obtained if we chose $\kappa_s = \Gamma(1-H)\ \Gamma(1/2+H)/[4^H\sqrt{\pi}]$

Simulations. To simulate a fBm constrained on its past, we used the Hosking algorithm [11, 12] in which the fBm is iteratively sampled at fixed time steps $t_i = n\Delta t$, each position $x(t_{i+1})$ being generated from $x(t_0 = 0), x(t_1), ..., x(t_i)$. To take into account the fact that the past trajectory is known, we defined a time t_m and replaced all positions $x(t_i)$ with $t_i < t_m$ by a starting position x_0. The next steps $x(t_j)$ with $t_j > t_m$ were generated according to the standard Hosking algorithm. In the limit $t_m \to \infty$, this corresponds to a fBm constrained on its past trajectory after removing its average value to obtain a symmetric (centered) process.

D. Detailed derivation of the equations of the formalism

1. *The link between the covariance of trajectories in the late future of the first-passage time and the persistence exponent [Derivation of Eq. (3)]*

Here, we consider a stochastic process in d dimensions, $\mathbf{x}(t) = (x(t), y(t), ...)$. We suppose it is Gaussian, so that it is defined by its mean value and its covariance. We also assume that it is isotropic, so that the mean value $\langle \mathbf{x}(t) \rangle$ is constant with time, and the covariance between coordinates takes the form $\text{Cov}[x_\alpha(t), x_\beta(t')] = \delta_{\alpha\beta}\sigma_0(t,t')$, where α, β label the different spatial coordinates. This is due to the fact that $\text{Cov}[x_\alpha(t), x_\beta(t')]$ is, at fixed t, t', an isotropic rank-2 tensor and is therefore proportional to $\delta_{\alpha\beta}$. We further assume that each coordinate $x_\alpha(t)$ is a Gaussian stochastic process that has long time stationary increments, so that σ_0 satisfies the scaling behavior (S1) with the condition (S4). If $d = 1$, we consider the first-passage problem to a fixed threshold, for which the persistence $S(t)$, i.e. the probability of not having crossed this threshold, decays as $S(t) \propto 1/t^\theta$. The natural generalization of this definition to $d \geq 1$ is to consider the first-passage problem to a target region (which is reduced to a point for $d = 1$); here we assume that the process is *compact* ($dH < 1$), so that the target can be taken as punctual without any ambiguity. We also assume the process is *non-smooth.* Our starting point is a generalized form of the renewal equation

$$p(\mathbf{0},t) = \int_0^t d\tau\ f(\tau) p(\mathbf{0},t|\text{FPT} = \tau), \tag{S25}$$

where $p(\mathbf{x},t)$ is the probability density to observe the position $\mathbf{x}$ (in d dimensions) at time t, starting from the initial conditions at $t = 0$, and $f(t) = -\partial_t S(t)$ is the probability density that the FPT is equal to t. Here we have used the hypothesis that the process is rough, and that it is compact, so that a punctual target can be found by the random walker. The above equation is simply obtained by partitioning the event of being inside the target at time t over the value of the first-passage time.

We define $p_\pi(x,t)$ as the probability density to observe $\mathbf{x}$ at a time t after the FPT:

$$p_\pi(\mathbf{x},t) \equiv \int_0^\infty d\tau\ p(\mathbf{x}, t+\tau|\text{FPT} = \tau) f(\tau). \tag{S26}$$

Next, we consider a fixed time T, and integrate the renewal equation (S25) between $t = 0$ and $t = T$:

$$\int_0^T dt\ p(\mathbf{0},t) = \int_0^T dt \int_0^t d\tau f(\tau) p(\mathbf{0},t|\text{FPT} = \tau) = \int_0^T d\tau \int_\tau^T dt f(\tau) p(\mathbf{0},t|\text{FPT} = \tau), \tag{S27}$$

where we have simply inverted the order of integration in the second equality. Setting $t = u + \tau$, we obtain

$$\int_0^T dt\ p(\mathbf{0},t) = \int_0^T d\tau \int_0^{T-\tau} du f(\tau) p(\mathbf{0},\tau+u|\text{FPT} = \tau) = \int_0^T du \int_0^{T-u} d\tau f(\tau) p(\mathbf{0},\tau+u|\text{FPT} = \tau), \tag{S28}$$

where, again the order of integration has been inverted in the second equality. Now, we integrate Eq. (S26) over t between $t = 0$ and $t = T$ and use Eq. (S28) to obtain

$$\int_0^T dt\ [p_\pi(\mathbf{0},t) - p(\mathbf{0},t)] = \int_0^T du \int_{T-u}^{\infty} d\tau f(\tau) p(\mathbf{0}, \tau + u|\text{FPT} = \tau). \tag{S29}$$

We will evaluate both sides of this equation for large T. To evaluate the right hand side (rhs) term, we assume that

$$p(\mathbf{0}, \tau + u|\text{FPT} = \tau) \sim B(\tau/u)/u^{dH} \qquad (u \to \infty, \tau \to \infty), \tag{S30}$$

where B is a scaling function that is assumed to be finite and admit finite limits for small and large arguments. The above hypothesis is reasonable, because in the future u of the FPT, one expects that the probability distribution of $\mathbf{x}$ will be some function whose extension is proportional $L \sim u^H$, so that the probability to be at $\mathbf{x} = \mathbf{0}$ is $\sim 1/L^d = 1/u^{dH}$. The prefactor that can depend on u/τ, and the hypothesis that B is finite is well supported by our simulations (see e.g. Fig. S1). Now, by definition of θ, we have for large times

$$f(t) \underset{t\to\infty}{\simeq} f_0/t^{\theta+1}, \tag{S31}$$

where f_0 is a prefactor. The term on the right hand side of Eq. (S29) can be evaluated in the large T limit by setting $\tilde{u} = u/T$ and $\tilde{\tau} = \tau/T$ and taking the large T limit in the resulting expression, leading to

$$\int_0^T du \int_{T-u}^{\infty} d\tau f(\tau) p(\mathbf{0}, \tau + u|\text{FPT} = \tau) = T^2 \int_0^1 d\tilde{u} \int_{1-\tilde{u}}^{\infty} d\tilde{\tau} f(T\tilde{\tau}) p(\mathbf{0}, T(\tilde{\tau} + \tilde{u})|\text{FPT} = T\tilde{\tau}) \tag{S32}$$

$$\underset{T\to\infty}{\simeq} T^{1-\theta-dH} \int_0^1 d\tilde{u}\ Q(\tilde{u}), \tag{S33}$$

where Q is given by

$$Q(\tilde{u}) = \int_{1-\tilde{u}}^{\infty} d\tilde{\tau} \frac{f_0 B(\tilde{\tau}/\tilde{u})}{\tilde{\tau}^{1+\theta} \tilde{u}^{dH}} = \frac{f_0}{\tilde{u}^{\theta+dH}} \int_{(1-\tilde{u})/\tilde{u}}^{\infty} d\xi \frac{B(\xi)}{\xi^{1+\theta}}. \tag{S34}$$

The existence of Q is guaranteed by the fact that $\theta > 0$. Its asymptotics read

$$Q(\tilde{u} \to 0) \simeq \frac{f_0}{\tilde{u}^{\theta+dH}} \frac{B(\infty)}{\theta \tilde{u}^{-\theta}} \simeq \frac{f_0 B(\infty)}{\theta\ \tilde{u}^{dH}}, \qquad Q(\tilde{u} \to 1) \simeq f_0 \frac{B(0)}{\theta\ (1-\tilde{u})^{\theta}}. \tag{S35}$$

The above relations suffice to establish that $\int_0^1 dx Q(x)$ exists if $\theta < 1$ and $dH < 1$. The condition $dH < 1$ was already assumed (compact process), and from now on we also assume $\theta < 1$ (meaning that the mean first-passage time is infinite). In these conditions, we deduce the scaling

$$\int_0^T du \int_{T-u}^{\infty} d\tau f(\tau) p(\mathbf{0}, \tau + u|\text{FPT} = \tau) \propto T^{1-\theta-dH}. \tag{S36}$$

We now evaluate the term in the left hand side of Eq. (S29). At leading order, dimensional analysis indicate that

$$p(\mathbf{0},t) \underset{t\to\infty}{\sim} \frac{K_0}{t^{dH}}, \qquad p(\mathbf{0},t) \underset{t\to\infty}{\sim} \frac{K_\pi}{t^{dH}}, \tag{S37}$$

where K_0 and K_π are constants. Since $dH < 1$, if $K_0 \neq K_\pi$, we have at leading order

$$\int_0^T dt\ [p_\pi(\mathbf{0},t) - p(\mathbf{0},t)] \underset{T\to\infty}{\sim} \frac{(K_\pi - K_0)}{1 - dH} T^{1-dH}. \tag{S38}$$

This behavior must be matched with that of the rhs of (S29), which behaves as $T^{1-dH-\theta}$ from Eq. (S36). Since $\theta > 0$, we obtain a contradiction, which means that at long times, $p(\mathbf{0},t)$ and $p_\pi(\mathbf{0},t)$ are equal at leading order:

$$K_0 = K_\pi. \tag{S39}$$

We thus postulate an exponent $\alpha > dH$ for the next-to-leading order correction, such that

$$p_\pi(\mathbf{0},t) - p(\mathbf{0},t) \simeq \frac{K_1}{t^\alpha} + ... \qquad (t \to \infty). \tag{S40}$$

In order to find the value of α, we have to discuss according to the value of θ. If $\theta < 1 - dH$, we see from Eq. (S36) that the rhs of Eq. (S29) diverges with T for large T. This means that $\alpha < 1$, so that the left hand side term of Eq. (S29) also diverges with T, as $T^{1-\alpha}$. Equating the divergences leads to

$$\alpha = dH + \theta. \tag{S41}$$

In the opposite case $\theta > 1 - dH$, we see from Eq. (S36) that the rhs of Eq. (S29) tends to 0 with T for large T. This means that the following condition must hold exactly:

$$\int_0^\infty dt\ [p_\pi(\mathbf{0},t) - p(\mathbf{0},t)] = 0 \qquad (\text{if } 1 > \theta > 1 - dH). \tag{S42}$$

Then, using the trick $\int_0^T(...) = \int_0^\infty(...) - \int_T^\infty(...)$, we find

$$-\int_T^\infty [p_\pi(\mathbf{0},t) - p(\mathbf{0},t)] \propto T^{1-dH-\theta}, \tag{S43}$$

which leads to $\alpha = dH + \theta$ again. To proceed further, we define the covariance in the future of the first-passage time,

$$\sigma_\pi(t,t') = \mathrm{Cov}[x(\mathrm{FPT}+t), x(\mathrm{FPT}+t')], \tag{S44}$$

and we define an exponent β characterizing the divergence of the mean square displacement (MSD) after the FPT as

$$\sigma_\pi(t,t) - \sigma_0(t,t) \simeq c_1\ t^\beta \qquad (t \to \infty). \tag{S45}$$

If we assume that the stochastic process in the future of the FPT is Gaussian (which is well supported by our simulations, see e.g. Fig. S1), we obtain $p_\pi(\mathbf{0},t) \propto \sigma_\pi(t,t)^{-d/2}$. This leads to $\alpha = (d+2)H - \beta$, and thus

$$\beta = 2H - \theta \tag{S46}$$

Inserting Eq. (S46) into Eq. (S45), we obtain Eq. (3) in the main text. In summary, here we have identified *a link between the persistence exponent θ and the exponent β which characterizes the MSD of the trajectories in the future of the FPT at long times.* Let us remind our hypotheses: (i) $dH < 1$ (compact process), (ii) $\theta < 1$ (infinite mean FPT), (iii) that the scaling function B characterizing the propagator in the future of the FPT is finite for small and large arguments, and (iv) the trajectories in the future of the FPT display Gaussian statistics. The validity of (iii) and (iv) can be appreciated on Fig. S1 in one example.

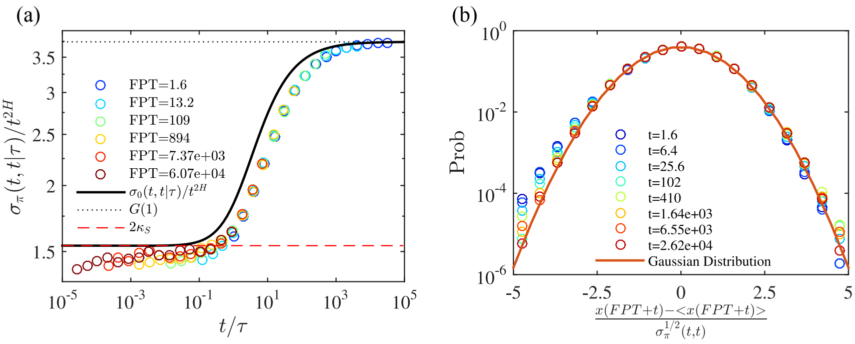

FIG. S1: Results obtained for type I process, for $H = 3/8$, $T = 10$ and $d = 1$. For these parameters, we have simulated numerically Eq. (S11), for which $x(t) = h(s = 0, t)$ starts at $x_0 = 1$. We have recorded the statistics of the trajectories after the first-passage to $x = 0$, and identified the conditional variance of $x(t + \mathrm{FPT})$ conditioned on the value of the $\mathrm{FPT} = \tau$. This quantity is represented in (a) in rescaled variables; if the Gaussian hypothesis holds then the function appearing B in Eq. (S30) is $B(y) = 1/\sqrt{2\pi\sigma_\pi(1,1|y)}$, hence these data support the fact that $B(y)$ is finite for $y \to \infty$ and $y \to 0$. The black line represents the ansatz B in Eq. (S79). In (b) we have represented the normalized distribution of $x(t + \mathrm{FPT})$ for various values of t, small deviations from Gaussian behavior can be observed at small t and disappear at larger values.

2. Self-consistent equations for the covariance of trajectories in the future of the FPT

We now write the generalization of the renewal equation (S25) for the probability to observe $\mathbf{x} = 0$ at t and the sequence of positions $\mathbf{x}_1, \mathbf{x}_2$ at times $t + t_1, t + t_2$, where t_1, t_2 are fixed times:

$$p(\mathbf{0}, t; \mathbf{x}_1, t + t_1; \mathbf{x}_2, t + t_2) = \int_0^t d\tau f(\tau)\ p(\mathbf{0}, t; \mathbf{x}_1, t + t_1; \mathbf{x}_2, t + t_2|\text{FPT} = \tau). \tag{S47}$$

We also define the probability density to observe the sequence $(\mathbf{0}; \mathbf{x}_1; \mathbf{x}_2)$ at respective times $t, t + t_1, t + t_2$ in the future of the FPT, averaged over the FPT distribution:

$$p_\pi(\mathbf{0}, t; \mathbf{x}_1, t + t_1; \mathbf{x}_2, t + t_2) = \int_0^\infty d\tau f(\tau) p(\mathbf{0}, t + \tau; \mathbf{x}_1, t + \tau + t_1; \mathbf{x}_2, t + \tau + t_2|\text{FPT} = \tau). \tag{S48}$$

Repeating the steps that had led to Eq. (S29), we obtain

$$\int_0^T dt[p_\pi(\mathbf{0}, t; \mathbf{x}_1, t + t_1; \mathbf{x}_2, t + t_2) - p(\mathbf{0}, t; \mathbf{x}_1, t + t_1; \mathbf{x}_2, t + t_2)] = \\ \int_0^T du \int_{T-u}^\infty d\tau f(\tau) p(\mathbf{0}, \tau + u; \mathbf{x}_1, u + \tau + t_1; \mathbf{x}_2, u + \tau + t_2|\text{FPT} = \tau). \tag{S49}$$

Here we assume, without loss of generality, that the process is centered (zero average) ; we stress that this has no consequence on the value of θ which does not depend on microscopic details. We multiply the above equation by $x_1 x_2$ and integrate over all positions to obtain the equation for the covariances:

$$\int_0^T dt\ [p_\pi(\mathbf{0}, t)\sigma^*_\pi(t_1, t_2|t) - p(\mathbf{0}, t)\sigma^*(t_1, t_2|t)] = \int_0^T du \int_{T-u}^\infty d\tau f(\tau) p(\mathbf{0}, \tau + u|\text{FPT} = \tau)\Sigma(t_1, t_2, u, \tau) \equiv C(t_1, t_2, T). \tag{S50}$$

Here, the term C is defined by this equation, and we have used the following notations for conditional covariances:

$$\sigma^*(t_1, t_2|t) = \text{Cov}(x(t + t_1), x(t + t_2)|x(t) = 0), \tag{S51}$$
$$\sigma^*_\pi(t_1, t_2|t) = \text{Cov}(x(\text{FPT} + t + t_1), x(\text{FPT} + t + t_2)|x(\text{FPT} + t) = 0), \tag{S52}$$
$$\Sigma(t_1, t_2, u, \tau) = \text{Cov}(x(u + \tau + t_1), x(u + \tau + t_2)|x(u + \tau) = 0, \text{FPT} = \tau). \tag{S53}$$

Now, the main problem consists in evaluating Σ, which is difficult since it depends it-self on the value of the first-passage time. Nevertheless, we argue below that it is reasonable to assume that, when a long time has ocured after the first-passage, this conditional covariance becomes equal to the long-time stationary covariance:

$$\Sigma(t_1, t_2, u, \tau) \sim \sigma_s(t_1, t_2) \qquad\qquad (\tau, u \to \infty). \tag{S54}$$

Here, we consider the above equality as an hypothesis, whose validity will be discussed later (see Section E). If Eq. (S54) holds, then the term C can be evaluated in the limit $T \to \infty$ as

$$C(t_1, t_2, T) = T^2 \int_0^1 d\tilde{u} \int_{1-\tilde{u}}^\infty d\tilde{\tau} f(T\tilde{\tau}) p(\mathbf{0}, T[\tilde{\tau} + \tilde{u}|\text{FPT} = T\tilde{\tau})\Sigma(t_1, t_2, \tilde{u}T, \tilde{\tau}T) \\ \underset{T\to\infty}{\sim} T^{1-\theta-dH} \int_0^1 d\tilde{u} \int_{1-\tilde{u}}^\infty d\tilde{\tau} \frac{f_0 B(\tilde{\tau}/\tilde{u})}{\tilde{\tau}^{1+\theta}\tilde{u}^{dH}} \sigma_s(t_1, t_2), \tag{S55}$$

with the same notations as in Eq. (S33). What is important here is the scaling with T. We see that if $\theta > 1 - dH$, C tends to zero in the large T limit, this suggests that in the large T limit, Eq. (S50) becomes

$$\int_0^\infty dt\ [p_\pi(\mathbf{0}, t)\sigma^*_\pi(t_1, t_2|t) - p(\mathbf{0}, t)\sigma^*(t_1, t_2|t)] = 0. \tag{S56}$$

This equation can be written explicitly as a function of the covariances σ_0 and σ_π:

$$\mathcal{H}(\tau, \tau') \equiv \int_0^\infty dt \left\{ \frac{\left[\sigma_\pi(t + \tau, t + \tau') - \frac{\sigma_\pi(t+\tau,t)\sigma_\pi(t+\tau',t)}{\sigma_\pi(t,t)}\right]}{\sigma_\pi(t, t)^{d/2}} - \frac{\left[\sigma_0(t + \tau, t + \tau') - \frac{\sigma_0(t+\tau,t)\sigma_0(t+\tau',t)}{\sigma_0(t,t)}\right]}{\sigma_0(t, t)^{d/2}} \right\} = 0. \tag{S57}$$

This equation defines the covariance matrix for $\theta > 1 - dH$. However, it cannot be true for $\theta < 1 - dH$ since the above integral does not exist. Let us consider now the case $\theta < 1 - dH$, for which we see that C diverges for large T [Eq. (S55)]. Our approach consists in combining Eqs. (S50) and Eq (S29) [multiplied by $\sigma_s(t_1,t_2)$] to obtain

$$\int_0^T dt\{p_\pi(\mathbf{0},t)[\sigma_\pi^*(t_1,t_2,t)-\sigma_s(t_1,t_2)] - p(\mathbf{0},t)[\sigma^*(t_1,t_2|t) - \sigma_s(t_1,t_2)]\}$$
$$= \int_0^T du \int_{T-u}^\infty d\tau f(\tau) p(\mathbf{0},\tau+u|\text{FPT}=\tau)[\Sigma(t_1,t_2,u,\tau) - \sigma_s(t_1,t_2)] \equiv R(t_1,t_2,T), \quad \text{(S58)}$$

where R is defined as the rhs of this equation. We note that R is no longer of order $T^{1-dH-\theta}$, this problematic term has been removed. Assuming that R vanishes for large T (this will be discussed in Section E), we obtain:

$$\mathcal{H}(\tau,\tau') \equiv \int_0^\infty dt \Bigg\{ \frac{1}{\sigma_\pi(t,t)^{d/2}} \left[\sigma_\pi(t+\tau,t+\tau') - \frac{\sigma_\pi(t+\tau,t)\sigma_\pi(t+\tau',t)}{\sigma_\pi(t,t)} - \sigma_s(\tau,\tau') \right]$$
$$- \frac{1}{\sigma_0(t,t)^{d/2}} \left[\sigma_0(t+\tau,t+\tau') - \frac{\sigma_0(t+\tau,t)\sigma_0(t+\tau',t)}{\sigma_0(t,t)} - \sigma_s(\tau,\tau') \right] \Bigg\} = 0. \quad \text{(S59)}$$

This equation is valid for $\theta < 1 - dH$; in fact it is also correct for $\theta > 1 - dH$, as can be seen by combining Eqs. (S57) and (S42). For $\theta = 1 - dH$ and $d = 1$, we recover the equation for processes with stationary increments [13]. In summary, here we have obtained self-consistent equations [Eqs. (S57) and (S59)] for the covariance in the future of the first-passage time for non-stationary Gaussian processes.

3. *Equation for the large time behavior of the trajectories after the first-passage [Derivation of Eq. (4)]*

Up to now, the equations for $\sigma_\pi(t,t')$ involve all time scales of the process, including microscopic ones. Here, our goal is to isolate the contribution of large times. First, we note that it is natural to assume that $\sigma_\pi(t,t') \sim t^{2H} G_\pi(t/t')$ for large t,t'. We see that $\mathcal{H}(\tau,\tau')$ in Eqs. (S57) and (S59) is proportional to $\tau^{1-(d-2)H}$ multiplied by a scaling function of τ/τ', and the fact that this term must vanish imposes that $G_\pi(x) = G(x)$ for all x. This equality generalizes the result (S39). Therefore, we look at large time corrections for σ_π, of the type

$$\sigma_\pi(t,t') \simeq \sigma_0(t,t') + \rho(t,t') + \ldots \qquad \rho(t,t') = t^{2H-\theta} z(t/t'), \quad \text{(S60)}$$

with z a scaling function. In the following, we look for an equation that defines the function $z(x)$.

First, we consider the case $\theta < 1 - dH$, for which σ_π satisfies Eq. (S59), which we write for $\tau = Tv$ and $\tau' = Tv'$:

$$\mathcal{H}(Tv,Tv') = \int_0^\infty du \Bigg\{ \frac{T}{\sigma_\pi(Tu,Tu)^{d/2}} \left[\sigma_\pi(T(u+v),T(u+v')) - \frac{\sigma_\pi(T(u+v),Tu)\sigma_\pi(T(u+v'),Tu)}{\sigma_\pi(Tu,Tu)} - \sigma_s(Tv,Tv') \right]$$
$$- \frac{T}{\sigma_0(Tu,Tu)^{d/2}} \left[\sigma_0(T(u+v),T(u+v')) - \frac{\sigma_0(T(u+v),Tu)\sigma_0(T(u+v'),Tu)}{\sigma_0(Tu,Tu)} - \sigma_s(Tv,Tv') \right] \Bigg\} = 0, \quad \text{(S61)}$$

where we have set $t = Tu$. Using (S60), the evaluation of all terms when $T \to \infty$ leads to

$$\mathcal{H}(Tv,Tv') \underset{T\to\infty}{\sim} T^{1-dH+2H-\theta} \int_0^\infty \frac{du}{\sigma(u,u)^{d/2}} \Bigg\{ \rho(u+v,u+v') - \frac{\sigma(u+v,u)\rho(u+v',u)}{\sigma(u,u)} - \frac{\sigma(u+v',u)\rho(u+v,u)}{\sigma(u,u)}$$
$$+ \rho(u,u)\frac{\sigma(u+v,u)\sigma(u+v',u)}{\sigma(u,u)^2} - \frac{d}{2}\frac{\rho(u,u)}{\sigma(u,u)} \left[\sigma(u+v,u+v') - \frac{\sigma(u+v,u)\sigma(u+v',u)}{\sigma(u,u)} - \sigma_s(v,v') \right] \Bigg\} = 0. \quad \text{(S62)}$$

It can be checked that this integral exists for $\theta < 1 - dH$. In particular, the integrand of the above expression for small u is $\sim [\sigma(v,v') - \sigma_s(v,v')]/u^{dH+\theta}$, which is integrable since we have $\theta < 1 - dH$. Since $\mathcal{H}$ must vanish at all orders, equating the above expression to zero gives Eq. (4) of the main text, that defines $\rho(v,v')$ for $\theta < 1 - dH$.

If $\theta > 1 - dH$, we follow the same steps as for $\theta < 1 - dH$, but we start from Eq. (S57) [instead of (S59)]. We obtain

$$\mathcal{H}(Tv, Tv') \underset{T\to\infty}{\sim} T^{1-dH+2H-\theta} \int_0^\infty \frac{du}{\sigma(u,u)^{d/2}} \Bigg\{ \rho(u+v, u+v') - \frac{\sigma(u+v,u)\rho(u+v',u)}{\sigma(u,u)} - \frac{\sigma(u+v',u)\rho(u+v,u)}{\sigma(u,u)} + \rho(u,u)\frac{\sigma(u+v,u)\sigma(u+v',u)}{\sigma(u,u)^2} - \frac{d}{2}\frac{\rho(u,u)}{\sigma(u,u)} \left[\sigma(u+v,u+v') - \frac{\sigma(u+v,u)\sigma(u+v',u)}{\sigma(u,u)} - \sigma(v,v') \right] \Bigg\} = 0. \quad \text{(S63)}$$

Note that the only difference with Eq. (S62) is the last term, where σ appears in place of σ_s. For $u \to \infty$, the integrand of the above expression is $\sim d[\sigma_s(v,v') - \sigma(v,v')]/(2u^{dH+\theta})$, which is integrable since $\theta > 1 - dH$. Equating the above expression to zero leads to Eq. (4) in the main text for $\theta > 1 - dH$.

4. *The equation for $z(x)$ [Derivation of Eq. (6)]*

Due to scaling properties, the solutions of Eqs. (S62) and (S63) can be found under the scaling form $\rho(v,v') = v^{2H-\theta} z(v/v')$, where the single-valued function $z(x)$ satisfies a linear equation which we determine now. Using the same arguments as in Section A, we can show that, if the process in the future of the FPT has long-time stationary increments, and if $z(x)$ has a linear term in its expansion near $x = 1$, then

$$z(x) \underset{x\to 1}{\simeq} z(1)[1 + \beta/2(1-x)] + ... \quad \text{(S64)}$$

Since it is reasonable to believe that the process in the future of the FPT admits stationary increments at long times, it is also reasonable to assume the above equality holds, and it is actually the case for the functions z determined at perturbative order, see Eqs. (S105),(S126). This suggests to pose

$$w(x) = \frac{z(x)}{z(1)} - \left[1 + \frac{\beta}{2}(1-x) \right], \quad \text{(S65)}$$

so that $w(1) = 0$. At this stage, we note that it is perfectly equivalent to look for $w(x)$ or for $z(x)$, and we will see later that many difficulties can be overcome by using $w(x)$ instead of $z(x)$. The equation for $w(x)$ is obtained by inserting Eqs. (S60) and (S65) into Eqs. (S62) and (S63), in which we also set $v = 1$ and $v' = 1/y$, with $0 < y < 1$. Splitting the resulting integral into a term proportional to $w(x)$ and a second member, we obtain

$$\int_0^\infty \frac{du}{u^{dH}} \left\{ (u+1)^\beta w\left(\frac{u+1}{u+y^{-1}}\right) - \frac{u^\beta}{G(1)} \left[G\left(\frac{u}{u+1}\right) w\left(\frac{u}{u+y^{-1}}\right) + G\left(\frac{u}{u+y^{-1}}\right) w\left(\frac{u}{u+1}\right) \right] \right\} = f(y), \quad \text{(S66)}$$

where the second member $f(y)$ reads

$$f(y) = \int_0^\infty du \; I_f(u,y), \quad \text{(S67)}$$

$$I_f(u,y) = \frac{(-1)}{u^{dH}} \Bigg\{ (u+1)^\beta \left(1 + \frac{\beta(1-y)}{2(uy+1)}\right) - \frac{u^\beta}{G(1)} \left[G\left(\frac{u}{u+1}\right)\left(1 + \frac{\beta}{2(uy+1)}\right) + G\left(\frac{uy}{uy+1}\right)\left(1 + \frac{\beta}{2(u+1)}\right) \right] + \frac{u^\beta}{G(1)^2} G\left(\frac{u}{u+1/y}\right) G\left(\frac{u}{u+1}\right)\left(1 + \frac{d}{2}\right) - \frac{d}{2u^\theta G(1)} \left[(u+1)^{2H} G\left(\frac{u+1}{u+1/y}\right) - G_K(y) \right] \Bigg\}, \quad \text{(S68)}$$

with the notation

$$G_K(y) = \begin{cases} G_s(y) & \text{if } \theta < 1 - dH, \\ G(y) & \text{if } \theta > 1 - dH. \end{cases} \quad \text{(S69)}$$

Eq. (S66) can be written under the simpler form

$$\int_0^1 dx \; K(x,y) \; w(x) = f(y) \quad \text{(S70)}$$

where the kernel $K(x,y)$ is identified by separating the integrals for the 3 terms in the left-hand side of Eq. (S66) and performing a change of variable in each of them, so that x corresponds to the argument of w (i.e. $x=(u_1+1)/(u_1+1/y)$ in the first term, $x=u_2/(u_2+1/y)$ in the second one and $x=u_3/(u_3+1)$ in the third one). This leads to

$$K(x,y)=H(x-y)\frac{du_1}{dx}\frac{(u_1+1)^{2H-\theta}}{u_1^{dH}}-\frac{du_2}{dx}\frac{u_2^{2H-\theta}}{u_2^{dH}G(1)}G\left(\frac{u_2}{u_2+1}\right)-\frac{du_3}{dx}\frac{u_3^{2H-\theta}}{u_3^{dH}G(1)}G\left(\frac{u_3}{u_3+1/y}\right), \tag{S71}$$

where $H(x)$ is the Heaviside step function, and

$$u_1=\frac{x-y}{(1-x)y},\qquad u_2=\frac{x}{(1-x)y},\qquad u_3=\frac{x}{1-x}. \tag{S72}$$

Technical note for the numerical evaluation of $f(y)$. Of note, evaluating numerically $f(y)$ is not straightforward because the integrand $I_f(u,y)$ does not always converge rapidly to zero for large u (depending on the value of θ, H, d...) and a similar problem arises for small u. The strategy to evaluate f numerically consists in identifying analytically the behavior of f for small/large arguments and using this knowledge in the numerical program. More precisely, we identify the first terms in the expansion of $I_f(u,y)$ as

$$I_f(y,u)\underset{u\to\infty}{\simeq}\frac{a_1^+}{u^{\nu_1^+}}+\frac{a_2^+}{u^{\nu_2^+}}+\frac{a_3^+}{u^{\nu_3^+}}+...,\qquad I_f(y,u)\underset{u\to 0}{\simeq}\frac{a_1^-}{u^{\nu_1^-}}+\frac{a_2^-}{u^{\nu_2^-}}+\frac{a_3^-}{u^{\nu_3^-}}+... \tag{S73}$$

Then, $f(y)$ can be evaluated as follows:

$$f(y)=\int_0^1 du\left(I_f(y,u)-\sum_{i=1}^{n^-}\frac{a_i^-}{u^{\nu_i^-}}\right)+\int_1^\infty du\left(I_f(y,u)-\sum_{i=1}^{n^+}\frac{a_i^+}{u^{\nu_i^+}}\right)+\sum_{i=1}^{n^-}\frac{a_i^-}{1-\nu_i^-}+\sum_{i=1}^{n^+}\frac{a_i^+}{\nu_i^+-1}, \tag{S74}$$

where n_+, n^- are the number of terms for which the expansion of I_f is analytically known, and where now the integrals converge rapidly. This procedure thus require to identify the first order terms of the expansion of $I_f(u,y)$. After some algebra, using the expansion (S4) for G, we obtain

$$\begin{cases}\nu_1^+=dH+\theta, & a_1^+=d[G_s(y)-G_K(y)]/[2G(1)]\\ \nu_2^+=dH+\theta+2H, & a_2^+=-\kappa_s^2\left(1+d/2\right)/G(1)^2y^{2H}\\ \nu_3^+=dH+\theta+1-2H, & a_3^+=0\\ \nu_4^+=dH+\theta+1, & a_4^+=\frac{\kappa_s}{G(1)}\left(\frac{1}{y^{2H}}+\frac{1}{y}\right)\left[\left(1+\frac{d}{2}\right)H-\frac{\beta}{2}\right]-\frac{d}{G(1)}H\kappa_s\left(1/y-1\right)^{2H}\end{cases}$$

and, for small arguments:

$$\begin{cases}\nu_1^-=dH+\theta, & a_1^-=d[G(y)-G_K(y)]/[2G(1)]\\ \nu_2^-=dH, & a_2^-=-\left(1+\beta(1-y)/2\right)\\ \nu_3^-=dH+\theta-2H, & a_3^-=2\frac{G(0)}{G(1)}(1+\beta/2)-\frac{G(0)^2}{G(1)^2}\left(1+\frac{d}{2}\right)\\ \nu_4^-=dH-2H+\theta-2\mu, & a_4^-=-\frac{\alpha_1^2}{G(1)^2}\left(1+\frac{d}{2}\right)y^\mu\\ \nu_5^-=dH-2H+\theta-\mu, & a_5^-=\frac{\alpha_1}{G(1)}\left(1+\frac{\beta}{2}\right)(1+y^\mu)-\frac{G_0\alpha_1}{G(1)^2}(1+y^\mu)\left(1+\frac{d}{2}\right)\end{cases}$$

where G_0, α_1, μ characterize the behavior of G near zero:

$$G(x)\underset{x\to 0}{\simeq}G_0+\alpha_1 x^\mu+...,\qquad \mu=\begin{cases}1-2H & \text{(type I)}\\ 1/2-H & \text{(type II)}\end{cases}$$

5. *Behavior of z for small arguments and the criterion to select the correct value of θ*

Numerical integration of $z(x)$ tends to show the presence of divergences for small values of x. Here, we give an argument to identify a possible exponent α such that

$$z(x)\underset{x\to 0}{\sim}\frac{1}{x^\alpha}. \tag{S75}$$

Let us look for non-trivial values of α, i.e. which are not contained in the expansions of $G(x)$. If one looks at Eq. (S66) for large y^{-1}, we will find terms $\propto y^{-\alpha}$ that cannot be compensated by any other term if α is not trivial. The coefficient for this term must then vanish, leading to

$$\int_0^\infty \frac{dt}{t^{dH}} \left\{ \frac{(t+\tau)^{2H-\theta}}{(t+\tau)^\alpha} - \frac{t^{2H-\theta}}{t^\alpha} \times \frac{G(t/(t+\tau))}{G(1)} \right\} y^{-\alpha} + ... = 0. \tag{S76}$$

The values of α that are authorized are those for which the prefactor of $y^{-\alpha}$ in the above equation vanish, this leads to

$$\alpha = \nu + 2H - \theta, \tag{S77}$$

where ν is an exponent that does not depend on θ and satisfies the equation

$$\int_0^\infty \frac{dt}{t^{dH}} \left\{ \frac{1}{(t+1)^\nu} - \frac{1}{t^\nu} \times \frac{G(t/(t+1))}{G(1)} \right\} = 0. \tag{S78}$$

When $G = G_s$ and $d = 1$, a solution is $\nu = 1 - 2H$ [13] and $\theta = 1 - H$ so that $\alpha = H$. This means that most solutions $z(x)$ will diverge for small arguments. We postulate that the criterion to select the correct value of θ is to look for solutions that do not present this divergence as $1/x^\alpha$.

Procedure for the numerical evaluation of θ. To evaluate θ, we have proceeded as follows: for a given "test" value of θ, and a given choice of mesh points y_i, we evaluated the numerical solution of Eq. (S70). We observed that the obtained values of $z(x)$ behaved as predicted by (S75),(S77) and defined a coefficient C_θ so that $z(x) \simeq C_\theta/x^{\nu+2H-\theta}$. Repeating the procedure for several "attempt" values of θ enables us to identify the value of θ for which $C_\theta = 0$; we chose this value as the output of our algorithm. We controlled that the results do not depend on the choice of the mesh.

E. Discussion of the validity of our hypotheses

Here, we discuss the validity of our theory. The first issue with our approach comes from the selection criterium of the correct value of θ via the argument that $z(x)$ (or, equivalently, $w(x)$) does not diverge at small values of x. This criterium seems plausible at least when $2H - \theta > 0$ in Eq. (S60), since for this value $\rho(t, t') \sim t^{2H-\theta}$ diverges for large t. However, this argument is less plausible when $2H - \theta < 0$. This is supported by Fig. S2, where we have compared the theoretical predictions of θ to the simulations for all the models presented in the main text, it is clearly seen that most deviations between theory and simulations appear when $\theta - 2H > 0$. We argue that a minimal criterium for our theory to work is to require that the condition $2H - \theta > 0$ is satisfied at least for the stationary case ($\sigma = \sigma_s$), for which $\theta = 1 - dH$. *This argument leads to the conclusion that we should restrict ourselves to the case $H > 1/(d+2)$.*

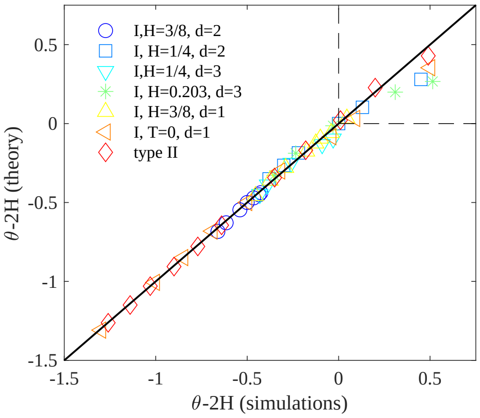

FIG. S2: Comparison of theoretical values of the persistence exponent versus simulations for all data shown in the main text.

Next, in Section D 2, we have derived an equation that defines σ_π. However, the relation (S54) was assumed to hold, and we also assumed that the right-hand-side of Eq. (S58) tends to zero. Here we discuss these hypotheses. Since

the evaluation of Σ requires the knowledge of the covariance after the first-passage, a quantity which is unknown, it seems necessary to make some ansatz to evaluate it. We have tried two ansatz:

$$\Sigma(t_1,t_2;u;\tau)\simeq\begin{cases}\sigma_\pi^*(t_1,t_2|u) & \text{(Ansatz A: decoupling approx.)}\\ \mathrm{Cov}[x(u+\tau+t_1),x(u+\tau+t_2)|x(u+\tau)=0;x(\tau)=0] & \text{(Ansatz B)}\end{cases}\tag{S79}$$

In the decoupling approximation (Ansatz A), one assumes that the covariance in the future of the FPT does not depend on the value of this FPT, this can be seen as a "mean-field" approximation whose validity can be evaluated self-consistently. This approximation also reminds us the independent interval approximation to calculate the duration of intervals between successive zeros of a smooth Gaussian process, for which the duration of an interval is assumed to be independent on the duration of the previous interval. In turn, Ansatz B consists in replacing the condition that $x=0$ was reached for the first time at τ by the simpler condition that $x=0$ is observed at τ (not necessarily for the first time). This kind of approximation has been proposed before in the different context of polymer cyclization [14]. It is motivated by the fact that it leads to rather accurate predictions for the mean square displacement in the future of the first-passage time, see Fig. S1.

In Ansatz A, we need to remember that $\sigma_\pi(t,t')\simeq\sigma_0(t,t')$ for large times, at leading order. It turns out that, in both ansatzs A and B, the property $\Sigma\to\sigma_s$ [Eq. (S54)] holds, which is an argument in favor of the validity of our equations. Next, we note that

$$\Sigma(t_1,t_2;u;\tau)\underset{\tau\to\infty}{\simeq}\begin{cases}\sigma_\pi(t_1,t_2|u) & \text{(Ansatz A)},\\ \sigma_s(t_1,t_2|u) & \text{(Ansatz B)}.\end{cases}\tag{S80}$$

This is enough to evaluate the rhs of Eq. (S58) as

$$R(t_1,t_2,T)\simeq T\int_0^T du\int_{1-u/T}^\infty d\tilde\tau f(T\tilde\tau)p(\mathbf{0},T\tilde\tau+u|\mathrm{FPT}=T\tilde\tau)[\Sigma(t_1,t_2,u,T\tilde\tau)-\sigma_s(t_1,t_2)]\tag{S81}$$

$$=T^{-\theta}\times\begin{cases}\int_0^\infty du\int_1^\infty d\tilde\tau\frac{f_0}{\tilde\tau^{1+\theta}}\frac{[\sigma_\pi(t_1,t_2|u)-\sigma_s(t_1,t_2)]}{[2\pi\sigma_0(u,u)]^{d/2}} & \text{(Ansatz A)}\\ \int_0^\infty du\int_1^\infty d\tilde\tau\frac{f_0}{\tilde\tau^{1+\theta}}\frac{[\sigma_s(t_1,t_2|u)-\sigma_s(t_1,t_2)]}{[2\pi\sigma_s(u,u)]^{d/2}} & \text{(Ansatz B)}\end{cases}\tag{S82}$$

In Eq. (S81) we have set $\tau=T\tilde\tau$ and the next evaluation follows from taking the limit $T\to\infty$ of all terms. The integrals in the above equation converge as soon as $\theta>0$, $dH<1$ (condition obtained for small u), and $(d+2)H>1$ (condition obtained for large u, for which $\sigma_s(t_1,t_2|u)-\sigma_s(t_1,t_2)\propto 1/u^{2H}$ from the property (S7), and the same holds for σ_π when one assumes that it has also long-time stationary increments). This means that, if $(d+2)H>1$, the leading order of the lhs of Eq. (S58) is $1/T^\theta$ for large T, which tends to zero, so that our equation for the covariance is correct (at least from the point of view of these investigations with our two ansatzs). Hence, we expect that our theory holds when $H>1/(d+2)$, this validity criterium is the same as the one found above.

In order to investigate the behavior of R for $H<1/(d+2)$, it is necessary to show that

$$\Sigma(t_1,t_2,u,\tau)\underset{u,\tau\to\infty}{\simeq}\begin{cases}\sigma_s(t_1,t_2)-\frac{\kappa_s^2(t_1t_2)^{2H}}{G(1)u^{2H}} & \text{(Ansatz A)},\\ \sigma_s(t_1,t_2)-\frac{\kappa_s^2\,(t_1t_2)^{2H}}{\sigma(u,u|\tau)} & \text{(Ansatz B)},\end{cases}\tag{S83}$$

where the result for Ansatz B comes from general formulas on conditional covariances [2] and the property (S7). If $(d+2)H<1$, we thus evaluate the rhs of Eq. (S58) as

$$R(t_1,t_2,T)=T^2\int_{1-\tilde u}^\infty d\tilde\tau f(T\tilde\tau)p(\mathbf{0},T\tilde\tau+T\tilde u|\mathrm{FPT}=T\tilde\tau)[\Sigma(t_1,t_2,T\tilde u,T\tilde\tau)-\sigma_s(t_1,t_2)]\tag{S84}$$

$$\simeq-\frac{T^{1-(d+2)H-\theta}}{(2\pi)^{d/2}}\kappa_s^2\times\begin{cases}\int_0^1 d\tilde u\int_{1-\tilde u}^\infty d\tilde\tau\frac{f_0}{\tilde\tau^{1+\theta}}\frac{1}{[G(1)u]^{(d+2)H}} & \text{(Ansatz A)},\\ \int_0^1 d\tilde u\int_{1-\tilde u}^\infty d\tilde\tau\frac{f_0}{\tilde\tau^{1+\theta}}\frac{1}{[\sigma(u,u|\tau)]^{d+2H}} & \text{(Ansatz B)}.\end{cases}\tag{S85}$$

These integrals exist when $0<\theta<1$ and $(d+2)H<1$ (which is assumed here), so that in this case $R\propto T^{1-(d+2)H-\theta}$. This means that when $\theta<1-(d+2)H$, our equation for the covariance is wrong since the rhs of (S58) does not vanish for large T. Since there is no reason why θ should be larger than $1-(d+2)H$, we consider that using our formalism for $(d+2)H<1$ is not advised.

In Fig. S3, we present simulation results for a value of H which does not satisfy this criterium ($H=1/4,d=1$). It is seen that the curve $\theta(T)$ is qualitatively correct, but there is a clear difference between the predicted value of θ

and the measured one. However, from the arguments above, we are unsure of our criterium as soon as $\theta > 2H = 1/2$, and we know that our equation for the covariance is wrong for $\theta < 1 - 3H = 0.25$. This leaves a very small range of values of θ in which the theory could be expected to be correct (which does not even include the stationary state $T = 1$) and this is probably the reason of the differences between theory and simulations.

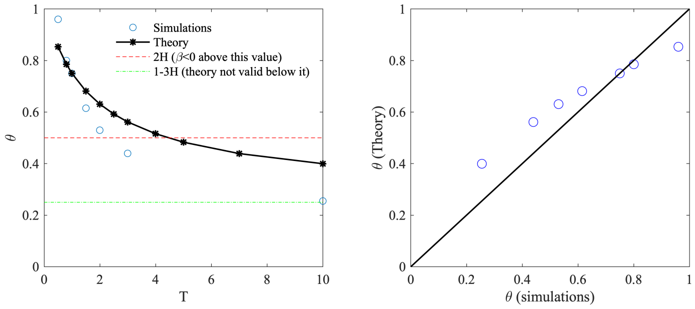

FIG. S3: Results for $H = 1/4$ and $d = 1$ (which lies outside the validity criterium $H > 1/(d + 2)$ of our theory, see text).

F. Perturbative analysis of the formalism [Derivation of Eqs. (7),(8)]

Here, we perform an analysis at perturbative order of our equations in dimension $d = 1$. We assume $H = 1/2 + \varepsilon$ with ε small and we start directly with the equation (S63) for $\rho(u, v)$. We assume $\theta > 1 - H$ (but we expect that the expansion in powers of ε will be the same for $\theta > 1 - H$ or $\theta < 1 - H$):

$$\int_0^\infty \frac{du}{u^H}\left\{\rho(u+v,u+v') - \frac{\sigma(u+v,u)\rho(u+v',u)}{\sigma(u,u)} - \frac{\sigma(u+v',u)\rho(u+v,u)}{\sigma(u,u)} + \rho(u,u)\frac{\sigma(u+v,u)\sigma(u+v',u)}{\sigma(u,u)^2}\right\}$$
$$= \int_0^\infty \frac{du}{2u^{H+\theta}}\left[\sigma(u+v,u+v') - \frac{\sigma(u+v,u)\sigma(u+v',u)}{\sigma(u,u)} - \sigma(v,v')\right] \equiv C(v,v'), \quad \text{(S86)}$$

where C is defined by this equation. Following our formalism, we will look for symmetric solutions with $\rho(u, u) = u^{2H-\theta}$ and that our criterium to select the correct value of θ is that $\rho(u, v)$ does not diverge with v as $v \to 0$. Let us write the expansion of θ as:

$$\theta = 1 - H + \varepsilon\delta_1 + \varepsilon^2\delta_2 + ..., \quad \text{(S87)}$$

with $\delta_1\varepsilon > 0$ to have $\theta > 1 - H$. Now, let us introduce the following notations

$$\sigma_s(t,t') = \min(t,t') + \varepsilon\sigma_{s,1}(t,t') + \varepsilon^2\sigma_{s,2}(t,t') + ... \qquad \sigma(t,t') = \sigma_s(t,t') + \varepsilon\omega_1(t,t') + \varepsilon^2\omega_2(t,t') + ... \quad \text{(S88)}$$
$$\rho(u,v) = \rho_0(u,v) + \varepsilon\rho_1(u,v) + \varepsilon^2\rho_2(u,v)... \quad \text{(S89)}$$

Here, ω_i represents the deviation of σ with respect to σ_s at order i. Note that the leading order term for ρ is of order zero because of the normalization $\rho(u, u) = u^{2H-\theta} \simeq \sqrt{u}$ when $H \to 1/2$.

1. *First order*

To estimate the limit of C for $\varepsilon \to 0$, let us introduce a parameter L (finite but large) and consider the calculation

$$C(v,v') = \int_0^L \frac{du}{2u^{H+\theta}}\left[\sigma(u+v,u+v') - \frac{\sigma(u+v,u)\sigma(u+v',u)}{\sigma(u,u)} - \sigma(v,v')\right] + \int_L^\infty \frac{du}{2u^{H+\theta}}\left[\sigma_s(v,v') - \sigma(v,v')\right],$$
$$= \int_0^L \frac{du}{2u^{H+\theta}}\left[\sigma(u+v,u+v') - \frac{\sigma(u+v,u)\sigma(u+v',u)}{\sigma(u,u)} - \sigma(v,v')\right] + \frac{L^{-\varepsilon\delta_1}}{2\varepsilon\delta_1}\left[\sigma_s(v,v') - \sigma(v,v')\right],$$
$$\underset{\varepsilon\to 0}{\simeq} -\frac{\omega_1(v,v')}{2\delta_1}. \quad \text{(S90)}$$

We thus obtain

$$\int_0^\infty \frac{du}{u^{1/2}} \left\{ \rho_0(u+v,u+v') - \rho_0(u+v',u) - \rho_0(u,u+v') + \rho_0(u,u) \right\} = -\frac{\omega_1(v,v')}{2\delta_1}. \tag{S91}$$

Let us take the derivative with respect to v and v' of this equation:

$$\int_0^\infty \frac{du}{u^{1/2}} \rho_0^{(1,1)}(u+v,u+v') = -\frac{\omega_1^{(1,1)}(v,v')}{2\delta_1}, \tag{S92}$$

with the notation $G^{(1,1)} = \partial_v \partial_{v'} G$ for all functions $G(v,v')$. The solution of this equation can be found by the method of superposition of solutions. Consider the simpler equation with a single complex exponential in the second member

$$\int_0^\infty \frac{du}{u^{1/2}} \rho_0^{(1,1)}(u+v,u+v') = e^{i(\omega v + \omega' v')}, \tag{S93}$$

for which an obvious solution is

$$\rho_0^{(1,1)}(v,v') = e^{i(\omega v+\omega' v')} \frac{1}{\int_0^\infty \frac{du}{u^{1/2}} e^{i(\omega+\omega')u}}. \tag{S94}$$

Introducing the double Fourier transform and its inverse for all functions $f(t,t')$ as

$$\hat{f}(\omega,\omega') = \int_{-\infty}^\infty dt \int_{-\infty}^\infty dt' e^{-i(\omega t+\omega' t')} f(t,t'), \qquad f(t,t') = \frac{1}{4\pi^2}\int_{-\infty}^\infty d\omega \int_{-\infty}^\infty d\omega' e^{i(\omega t+\omega' t')} \hat{f}(\omega,\omega'), \tag{S95}$$

we see that

$$\rho_0^{(1,1)}(v,v') = -\frac{1}{8\pi^2\delta_1}\int_{-\infty}^\infty d\omega \int_{-\infty}^\infty d\omega' \ \widehat{\omega_1^{(1,1)}}(\omega,\omega') e^{i(\omega v+\omega' v')} \frac{1}{\int_0^\infty \frac{du}{u^{1/2}} e^{i(\omega+\omega')u}} \tag{S96}$$

is a solution of (S92) constructed by superposition of solutions. Integrating with respect to v, v' and using $\rho_1(v,0) = \rho_1(0,v') = 0$, we obtain

$$\begin{aligned} \rho_0(\xi,\xi') &= \int_0^\xi dv \int_0^{\xi'} dv' \rho_0^{(1,1)}(v,v') \\ &= -\frac{1}{8\pi^2\delta_1}\int_{-\infty}^\infty d\omega \int_{-\infty}^\infty d\omega' \int_0^\infty dt \int_0^\infty dt' \omega_1^{(1,1)}(t,t') \frac{(e^{i\omega(\xi-t)} - e^{-i\omega t})(e^{i\omega'(\xi'-t')} - e^{-i\omega' t'})}{(-\omega\omega')} \frac{\sqrt{-i(\omega+\omega')}}{\sqrt{\pi}}. \end{aligned} \tag{S97}$$

We introduce a function $K_0(t,t')$ and its double Fourier transform

$$K_0(t,t') = \left(\frac{H(t'-t)}{\pi\sqrt{t}} + \frac{H(t-t')}{\pi\sqrt{t'}} \right) H(t)H(t'), \qquad \widehat{K}_0(\omega,\omega') = -\frac{\sqrt{i(\omega+\omega')}}{\omega\omega'\sqrt{\pi}}, \tag{S98}$$

with H the Heaviside step function. Therefore, if we change $\omega \to -\omega$ and $\omega' \to -\omega'$ in (S97) and integrate over ω, ω' before t, t', we recognize the double inverse Fourier transform of K and we obtain

$$\rho_0(\xi,\xi') = -\frac{1}{2\delta_1}\int_0^\infty dt \int_0^\infty dt' \omega_1^{(1,1)}(t,t') [K_0(t-\xi,t'-\xi') - K_0(t-\xi,t') - K_0(t,t'-\xi') + K_0(t,t')]. \tag{S99}$$

Now, using (S98) this equation can be integrated once [13], leading to

$$\rho_0(\xi,\xi') = \frac{1}{2\pi\delta_1}\int_0^\infty \frac{dt}{\sqrt{t}} [\omega_1^*(t+\xi,t+\xi') - \omega_1^*(t+\xi,t) - \omega_1^*(t,t+\xi') + \omega_1^*(t,t)], \tag{S100}$$

with ω_1^* is a symetrized first derivative of ω_1, i.e.

$$\omega_1^*(t,t') = (\partial_t + \partial_{t'})\omega_1(t,t'). \tag{S101}$$

Eq. (S100) is the solution of Eq. (S91). For the quenched fBm, we can calculate

$$\omega_1^*(t,t') = \lambda[2\ln(t+t') - \ln t - \ln t'], \tag{S102}$$

with $\lambda = 1 - T$. Inserting this value into Eq. (S100) and performing the integral, we obtain

$$\rho_0(\xi,\xi') = \frac{\lambda\sqrt{2}}{\delta_1}[\sqrt{\xi+\xi'} - \sqrt{\xi} - \sqrt{\xi'}]. \tag{S103}$$

Comparing the above equation with the property $\rho_0(\xi,\xi) = \sqrt{\xi}$ (which holds by the very definiton of ρ_0) enables us to identify δ_1:

$$\delta_1 = -2\lambda(\sqrt{2} - 1). \tag{S104}$$

This is consistent with (and generalizes) the results of Ref [3] for $\lambda = 1$ and $\lambda = 0$. It is also instructive to give the value of $z(x)$ at this order:

$$z(x) = -\frac{\sqrt{1+x} - \sqrt{1} - \sqrt{x}}{(2-\sqrt{2})\sqrt{x}}. \tag{S105}$$

2. *Second order (quenched fBm)*

At second order, we need the next-to-leading order of the expansion of the term C in Eq. (S86):

$$\begin{aligned}
C(v,v') &= \int_0^L \frac{du}{2u^{1+\varepsilon\delta_1}}\left[\sigma(u+v,u+v') - \frac{\sigma(u+v,u)\sigma(u+v',u)}{\sigma(u,u)} - \sigma(v,v')\right] + \frac{L^{-\varepsilon\delta_1-\varepsilon^2\delta_2}}{2[\varepsilon\delta_1+\varepsilon^2\delta_2]}\left[\sigma_s(v,v') - \sigma(v,v')\right] \\
&\simeq \varepsilon\int_0^L \frac{du}{2u}\left[\sigma_1(u+v,u+v') - \sigma_1(u+v,u) - \sigma_1(u,u+v') + \sigma_1(u,u) - \sigma_1(v,v')\right] + \frac{1-\varepsilon\delta_1\ln L}{2\varepsilon\delta_1(1+\varepsilon\delta_2/\delta_1)}\left[-\varepsilon\omega_1 - \varepsilon^2\omega_2\right] \\
&\simeq -\frac{\omega_1(v,v')}{2\delta_1} + \varepsilon C_2
\end{aligned} \tag{S106}$$

with

$$\begin{aligned}
C_2(v,v') = &\int_0^L \frac{du}{2u}\left[\omega_1(u+v,u+v') - \omega_1(u+v,u) - \omega_1(u,u+v') + \omega_1(u,u) - \omega_1(v,v')\right] \\
&+ \frac{\omega_1(v,v')\ln L}{2} + \omega_1(v,v')\frac{\delta_2}{2\delta_1^2} - \frac{\omega_2(v,v')}{2\delta_1}
\end{aligned} \tag{S107}$$

Using $\ln L = \int_1^L du/u$ in the above expression, we see that C_2 is independent of L and reads

$$\begin{aligned}
C_2(v,v') = &\int_0^1 \frac{du}{2u}\left[\omega_1(u+v,u+v') - \omega_1(u+v,u) - \omega_1(u,u+v') + \omega_1(u,u) - \omega_1(v,v')\right] + \\
&\int_1^\infty \frac{du}{2u}\left[\omega_1(u+v,u+v') - \omega_1(u+v,u) - \omega_1(u,u+v') + \omega_1(u,u)\right] + \omega_1(v,v')\frac{\delta_2}{2\delta_1^2} - \frac{\omega_2(v,v')}{2\delta_1}.
\end{aligned} \tag{S108}$$

Expanding Eq. (S86) at second order, we find that the equation for ρ_1 is

$$\int_0^\infty \frac{du}{u^{1/2}}\left\{\rho_1(u+v,u+v') - \rho_1(u+v,u) - \rho_1(u,u+v') + \rho_1(u,u)\right\} = A_1 + A_2 + C_2 \equiv A(v,v') \tag{S109}$$

$$\begin{aligned}
A_1 &= \int_0^\infty \frac{du}{u^{3/2}}\Big\{[\sigma_1(u+v,u) - \sigma_1(u,u)][\rho_0(u+v',u) - \rho_0(u,u)] + [\sigma_1(u+v',u) - \sigma_1(u,u)][\rho_0(u+v,u) - \rho_0(u,u)]\Big\} \\
A_2 &= \int_0^\infty du\left(\frac{\ln u}{u^{1/2}}\right)\left\{\rho_0(u+v,u+v') - \rho_0(u+v,u) - \rho_0(u,u+v') + \rho_0(u,u)\right\}
\end{aligned} \tag{S110}$$

Eq. (S109) is the same integral equation that appeared at first order, its solution with no divergence at the origin is

$$\rho_1(\xi,\xi) = -\frac{1}{\pi}\int_0^\infty \frac{dt}{\sqrt{t}}[A^*(t+\xi,t+\xi) - 2A^*(t+\xi,t) + A^*(t,t)]. \tag{S111}$$

Performing the integrations, we obtain

$$\rho_1(\xi,\xi) = \sqrt{\xi}[\alpha + \Omega\ln\xi], \qquad \Omega = 3 + 2(-1+\sqrt{2})\lambda = 3 - \delta_1. \tag{S112}$$

$$\alpha = -4 + c + \frac{(1+\sqrt{2})\delta_2}{2\lambda} + \lambda(4 - 4\sqrt{2} + b - 6\ln 2 + \sqrt{2}\ln 4) - \sqrt{2}\ln 8 \tag{S113}$$

where the numerical constants b and c are defined by the integrals

$$c = -\frac{(2+\sqrt{2})}{\pi}\int_0^\infty \frac{d\tau}{\sqrt{\tau}}\int_0^\infty \frac{du}{u^{3/2}}\frac{\partial}{\partial\tau}\Big\{ \left(\sqrt{\tau+u} - \sqrt{1+\tau+u} - \sqrt{\tau+2u} + \sqrt{1+\tau+2u}\ \right)\times$$
$$[(u+\tau)\ln(u+\tau) - \tau\ln\tau + (1+\tau)\ln(1+\tau) - (1+u+\tau)\ln(1+u+\tau)]\Big\} \tag{S114}$$

$$b = -\frac{2}{\pi(-2+\sqrt{2})}\int_0^\infty \frac{d\tau}{\sqrt{\tau}}\int_0^\infty \frac{du}{u^{3/2}}\frac{\partial}{\partial\tau}\Big\{ \left(\sqrt{\tau+u} - \sqrt{1+\tau+u} - \sqrt{\tau+2u} + \sqrt{1+\tau+2u}\ \right)\times$$
$$[(u+\tau)\ln(u+\tau) - (1+u+\tau)\ln(1+u+\tau) - (2u+\tau)\ln(2u+\tau) + (1+2u+\tau)\ln(1+2u+\tau)]\Big\} \tag{S115}$$

To comment on the value of Ω, let us remind that

$$\rho(\xi,\xi) = \xi^\beta = \xi^{2H-\theta} = \xi^{3H-1-\varepsilon\delta_1+\dots} = \sqrt{\xi} + \varepsilon(3-\delta_1)\sqrt{\xi}\ln\xi + \mathcal{O}(\varepsilon^2). \tag{S116}$$

If our theory is consistent the coefficient of $\varepsilon\sqrt{\xi}\ln\xi$ in the above equation is set by δ_1, whatever the value of δ_2. The fact that we find the same value with the above argument (S116) or by the complete calculation (S112) argues in favor of the consistency of our approach. We also see that there is no term $\varepsilon\sqrt{\xi}$ at first order in the above expression (S116), which imposes to set $\alpha = 0$; this equality can be satisfied by adjusting the value of δ_2. The result is

$$\delta_2 = a_1\lambda(a_2 + \lambda), \tag{S117}$$

with the constants

$$a_1 = -2(-1+\sqrt{2})(4 - 4\sqrt{2} + b - 6\ln 2 + \sqrt{2}\ln 4) \simeq 1.77, \qquad a_2 = \frac{-4 + c - \sqrt{2}\ln 8}{4 - 4\sqrt{2} + b - 6\ln 2 + \sqrt{2}\ln 4} \simeq 1.28, \tag{S118}$$

where the numerical values necessitate the numerical evaluation of the integrals in Eqs. (S114) and (S115). To summarize, our result at second order for the quenched fBm is

$$\theta = 1 - H + \varepsilon\delta_1 + \varepsilon^2\delta_2, \qquad \delta_1 = -2\lambda(\sqrt{2}-1) \qquad \delta_2 = 1.77\lambda(1.28+\lambda) \qquad \lambda = 1 - T. \tag{S119}$$

3. *Second order for the fBm constrained on its past*

For the fBm constrained on its past, we have again to evaluate the second member C in Eq. (S86), which reads:

$$C(v,v') = \int_0^L \frac{du}{2u^{H+\theta}}\left[\sigma(u+v,u+v') - \frac{\sigma(u+v,u)\sigma(u+v',u)}{\sigma(u,u)} - \sigma(v,v')\right] + \int_L^\infty \frac{du}{2u^{H+\theta}}\left[\sigma_s(v,v') - \sigma(v,v')\right]. \tag{S120}$$

Now, since $\sigma = \sigma_s$ at first order, and $\theta = 1 - H + \varepsilon^2\delta_2$, we get:

$$C(v,v') = \int_0^L \frac{du}{2u^{H+\theta}}\left[\sigma(u+v,u+v') - \frac{\sigma(u+v,u)\sigma(u+v',u)}{\sigma(u,u)} - \sigma(v,v')\right] + \frac{L^{-\varepsilon^2\delta_2}}{2\varepsilon^2\delta_2}\left[\sigma_s(v,v') - \sigma(v,v')\right]$$
$$\simeq -\frac{\omega_2(v,v')}{2\delta_2}. \tag{S121}$$

This means that the equation for ρ_0 is essentially the same as in the first order case, Eq. (S91) as soon as one replaces ω_1/δ_1 by ω_2/δ_2. From Eq. (S100) we deduce the solution for ρ_0

$$\rho_0(\xi,\xi') = \frac{1}{2\pi\delta_2}\int_0^\infty \frac{dt}{\sqrt{t}}[\omega_2^*(t+\xi,t+\xi') - \omega_2^*(t+\xi,t) - \omega_2^*(t,t+\xi') + \omega_2^*(t,t)]. \tag{S122}$$

We can identify ω_2 by expanding (S24) at second order

$$\omega_2(t,t') = -\int_0^\infty dx \frac{(t+xt')\ln(t+xt') - t\ln t - (t'x)\ln(t'x)}{x(1+x)}. \tag{S123}$$

The integral can be performed after taking the derivatives with respect to t and t'

$$\omega_2^*(t,t') = (\partial_t + \partial_{t'})\omega_2 = -\frac{1}{6}(2\pi^2 + 3\ln^2(t'/t)). \tag{S124}$$

Inserting this value into Eq. (S122) leads to

$$\rho_0(\xi,\xi') = \frac{2}{\delta_2}\left[(\sqrt{\xi}+\sqrt{\xi'})\ln(\sqrt{\xi}+\sqrt{\xi'}) - \sqrt{\xi}\ln\sqrt{\xi} - \sqrt{\xi'}\ln\sqrt{\xi'}\right] = \frac{1}{4\pi\delta_2}\sqrt{\xi}\; z(\xi/\xi') \tag{S125}$$

with

$$z(x) = \frac{\pi}{\sqrt{x}}[8(1+\sqrt{x})\ln(1+\sqrt{x}) - 4\sqrt{x}\ln x] \tag{S126}$$

Strikingly we observe a logarithmic divergence of $z(x)$ around $x=0$, as $z(x) \propto 1 - 2\ln x$. Again, the argument $\rho_0(\xi,\xi) = \sqrt{\xi}$ enables us to identify the value of $\delta_2 = 4\ln 2$, and thus the result of our perturbation theory for the fBm constrained on its past reads

$$\theta \simeq 1 - H + (4\ln 2)\varepsilon^2 + ... \tag{S127}$$

G. Result in the limit of high initial temperature, $T=\infty$

Let us consider the quenched fBm in the limit $T\to\infty$, which corresponds physically to the case that the initial temperature is much larger than the temperature of the dynamics. Thus, this corresponds to models starting with a fully "disordered" state. We see that the limiting value of the scaling function becomes

$$G_\infty(u) \equiv G_{T=\infty}(u) = \frac{-(1+u)^{2H} + (1+u^{2H})}{[-2^{2H}+2]u^{2H}}. \tag{S128}$$

thus we see that the behavior of $G_{T=\infty}(u)$ near $u=1$ is regular. Hence, according to Eq. (S9), after the Lamperti transform we obtain a smooth Gaussian process whose density of zero crossing can be analyzed with the independent interval approximation. Denoting again by $a(T)$ the correlator of the process after Lamperti transform, $a_\infty(T) = e^{-HT}G_\infty(e^{-HT})$, the result of the IIA is that θ is the smallest positive number that satisfies the equation $F(-\theta)=0$, where

$$F(s) = 1 + \frac{\pi\, s}{2|a''_\infty(0)|}\left[1 - \frac{2s}{\pi}\int_0^\infty dT e^{-sT}\operatorname{asin}(a_\infty(T))\right]. \tag{S129}$$

The value of θ deduced from this approximation for $T=\infty$ are close to $\theta \simeq 0.11 \pm 0.01$ for all values of H. This means that the perturbation theory around $H=1/2$ will fail at high temperatures, since the weakly non-Markovian limit and the limit of large initial temperature cannot be inverted.

SUPPLEMENTARY REFERENCES

[1] Bray, A. J., Majumdar, S. N. & Schehr, G. Persistence and first-passage properties in nonequilibrium systems. *Adv. Phys.* **62**, 225–361 (2013).

[2] Eaton, M. L. *Multivariate Statistics, A Vector Space Approach*, vol. 53 (Institute of Mathematical Statistics Beachwood, Ohio, USA, 1983).
[3] Krug, J. *et al.* Persistence exponents for fluctuating interfaces. *Phys. Rev. E* **56**, 2702–2712 (1997).
[4] Krapivsky, P., Mallick, K. & Sadhu, T. Dynamical properties of single-file diffusion. *J. Stat. Mech.: Theor. Exp.* **2015**, P09007 (2015).
[5] Blumen, A., Von Ferber, C., Jurjiu, A. & Koslowski, T. Generalized vicsek fractals: Regular hyperbranched polymers. *Macromolecules* **37**, 638–650 (2004).
[6] Dolgushev, M., Guérin, T., Blumen, A., Bénichou, O. & Voituriez, R. Contact kinetics in fractal macromolecules. *Phys. Rev. Lett.* **115**, 208301 (2015).
[7] Yaglom, A. M. Correlation theory of processes with random stationary n th increments. *Matematicheskii Sbornik* **79**, 141–196 (1955).
[8] Gripenberg, G. & Norros, I. On the prediction of fractional brownian motion. *J. Appl. Prob* **33**, 400–410 (1996).
[9] Anh, V. V. & Inoue, A. Prediction of fractional brownian motion with hurst index less than 1/2. *Bulletin of the Australian Mathematical Society* **70**, 321–328 (2004).
[10] Inoue, A. & Anh, V. V. Prediction of fractional processes with long-range dependence. *Hokkaido Mathematical Journal* **41**, 157–183 (2012).
[11] Hosking, J. R. Modeling persistence in hydrological time series using fractional differencing. *Water resources research* **20**, 1898–1908 (1984).
[12] Dieker, T. *Simulation of fractional Brownian motion.* Master's thesis (2004).
[13] Guérin, T., Levernier, N., Bénichou, O. & Voituriez, R. Mean first-passage times of non-markovian random walkers in confinement. *Nature* **534**, 356–359 (2016).
[14] Sokolov, I. M. Cyclization of a polymer: first-passage problem for a non-markovian process. *Phys Rev Lett* **90**, 080601 (2003).
